# The role of ozone treatment as integrative medicine. An evidence and gap map

**DOI:** 10.3389/fpubh.2022.1112296

**Published:** 2023-01-16

**Authors:** Maria Emilia Gadelha Serra, José Baeza-Noci, Carmen Verônica Mendes Abdala, Marilia Moura Luvisotto, Charise Dallazem Bertol, Ana Paula Anzolin

**Affiliations:** ^1^Brazilian Society of Medical Ozone Therapy (SOBOM), São Paulo, Brazil; ^2^World Federation of Ozone Therapy (WFOT), Brescia, Italy; ^3^Latin American and Caribbean Center on Health Sciences (BIREME/OPAS/OMS), São Paulo, Brazil

**Keywords:** systematic reviews, Ozone Therapy, Medical Ozone Therapy, evidence gap map, complementary therapies

## Abstract

**Introduction:**

The Brazil has one of the largest public health systems in the world and in the 1980's, Traditional, Complementary and Integrative Medicine were introduced. In 2018, the treatment with ozone became a complementary integrative practice showing several benefits. However, its effectiveness needs to be researched. The objective of this evidence gap map is to describe contributions of Integrative Medicines-Ozone treatment in different clinical conditions, to promote evidence-based practice.

**Methods:**

We applied the methodology developed by Latin American and Caribbean Center on Health Sciences Information based on the 3iE evidence gap map. The EMBASE, PubMed and Virtual Health Library databases, using the MeSH and DeCS terms for the treatment with Ozone were used.

**Results:**

26 systematic reviews were characterized, distributed in a matrix containing 6 interventions (parenteral oxygen/ozone gas mixture; parenteral ozonated water; systemic routes; topical application ozonated water; topical oxygen/ozone gas mixture; and topical ozonated oil) and 55 outcomes (cancer, infection, inflammation, pain, quality of life, wound healing and adverse effects). 334 associations between intervention and outcome were observed, emphasizing the parenteral oxygen/ozone gas mixture intervention (192 associations, 57%).

**Conclusions:**

The evidence gap map presents an overview of contributions of Ozone treatment in controlling pain, infections, inflammation and wound healing, as well as increasing the quality of life, and it is directed to researchers and health professionals specialized in Ozone treatment. No serious adverse effects were related. Therefore, this treatment may be even more widely known as an integrative treatment, considering its low cost, efficiency and safety. Future studies should adopt economic impact assessments and the organization of health services.

## 1. Introduction

The Brazilian Unified Health System (SUS) is one of the largest public health systems in the world. It covers most outpatient and inpatient care in the country, which currently has a population of over 190 million (1).

Since 1980 the experiences of Traditional, Complementary and Integrative Medicine (TCI) were included in the SUS in Brazil. In 2006, with the enactment of the National Policy on Integrative and Complementary Practices (PNPIC), the TCI gained visibility. In 2018, the PNPIC made official 25 other practices: art therapy, Ayurveda, biodance, circle dance, meditation, music therapy, naturopathy, osteopathy, chiropractic, reflex therapy, Reiki, shantala, integrative community therapy, yoga, apitherapy, aromatherapy, bioenergetics, family constellation, manual therapy, floral therapy and Ozone Therapy (2, 3).

Ozone is an oxygen triatome with high oxididant power. Ozone has been used for several purposes, including the treatment with Ozone, which is the therapeutic administration of medicinal ozone for the treatment of various diseases (4). This treatment is currently available and recognized as a medical procedure in many countries such as Germany, Italy, Russia, Portugal, Spain, Turkey, Greece, Egypt, China, Cuba, Mexico, Honduras and several Eastern European countries (4).

The treatment with ozone can be administered by different routes according to the targeted therapeutic purpose, considering a safety and efficacy “therapeutic window” that ranges from concentrations of 1μg/mL up to 100 μg/mL (5). This treatment has been used as an alternative for the treatment of osteoarthrosis (OA) (6, 7), wound healing (8–10) and low back pain (11, 12). However, the results of existing analyzes on the effectiveness and adverse events of ozone deserves to be better explored, bringing security to the user and prescriber of the treatment with ozone.

Evidence and Gaps Maps (EGMs) are an innovative quick review method that involves a systematic search on a topic of interest, identifying scientific evidence, trends, gaps in the knowledge and needs of future research (13). EGMs allow descriptive and visual analysis of the database, such as bubble graphs, to identify research gaps, to support evidence-based policy and to provide resources in clinical decision-making. Therefore, evidence maps, unlike other synthesis methods, use graphical representations (or dynamic representations, through interactive online databases), which facilitate the interpretation of results (14–16).

The World Health Organization (WHO) has encouraging and strengthening the insertion, recognition and use of integrative medicine. In this way, the Brazilian Society of Medical Ozone Therapy (SOBOM), World Federation of Ozone Therapy (WFOT) and the Latin-American and Caribbean Center on Health Sciences Information (BIREME/PAHO/WHO) have joined efforts to recruit volunteer researchers from world, to systematize the available scientific evidence. The objective of this EGMs is to describe contributions of Integrative Medicines-Ozone treatment in different clinical conditions, to promote evidence-based practice.

## 2. Methods

This EGMs summarizes interventions and health outcomes related to the effects the treatment with Ozone applied in different clinical conditions. We report the method and results according to PRISMA guidelines and the International Initiative for Impact Evaluation (3iE) Evidence Gap Methodology (13, 17). This EGMs was supported by a technical expert panel of librarians, practitioners, policy maker and researcher content experts.

### 2.1. Data sources

We searched PUBMED and EMBASE and Virtual Health Library (BVS) from 2006 to April 2022. BVS is a decentralized and dynamic collection of information sources on health. BVS is maintained by BIREME, a PAHO Specialized Center. This collection includes databases such as LILACS, MEDLINE, Cochrane Library and SciELO. We consulted topic experts and developed the search strategy together with BIREME.

A search strategy was developed, using the MeSH and DeCS terms ozonioterapia, ozonetherapy, Ozone Therapy and medical ozone.

The terms used in the search strategy were reviewed by treatment with ozone experts and researchers, and by librarians.

### 2.2. Inclusion criteria

Systematic Review (SR) were selected from randomized and non-randomized clinical trials, observational studies, in any language or publication date (2006–2022) considering the clinical application of OT, in Supplementary File Appendix S1.

### 2.3. Exclusion criteria

Primary non-review studies that did not include treatment with ozone as a clinical medical option, treatment with ozone applied in dentistry, methodological flaw and one study in mandarin were excluded, as shown in Supplementary File Appendix S2.

### 2.4. Risk of bias and data extraction

All reviews meeting the inclusion criteria were imported to an software Rayyan. After duplicates were removed, titles and abstracts were assessed for eligibility prior to full text review. Reviews fulfilling the inclusion criteria through full text review were then assessed for their quality. Quality was assessed using Assessment of Multiple Systematic Reviews (AMSTAR) checklist scores. The checklist contains 11 indicators that are used to derive an overall score assessed as high quality (score ≥ 8), medium quality (score 4–7), and low quality (score ≤ 3). Two reviewers (MEGS and APA) independently assessed the quality of each review.

Applying the AMSTAR tool, the SR were classified as: 1- green dot - high level of confidence, 2- yellow dot - moderate level of confidence and 3- red dot - low level of confidence and the according to the number of scientific studies, the dots received different sizes, distributed in each correspondent Outcome vs. Routes of administration. Bigger dots were related to 3 or more studies, medium dots were related to 2 studies and small dots had only 1 study.

### 2.5. Data synthesis

From each included study, we extracted the routes of administration (e.g., rectal insufflation, bag, ozonized oil, Minor Autohemotherapy) (Table 1) and the main health outcomes (e.g., inflammation, infection, pain, quality of life, side events, cancer, wound healing) that were summarized across included studies. Researchers and health professionals capacitated in the use of treatment with ozone in the management of outcomes and symptoms, especially in the dimension of disease and side events analyzed the data.

**Table 1:** Routes of administration of the oxygen-ozone gas mixture.

**Interventions group**	**Interventions**
Enteral ozonated oil	Ingestion
Enteral ozonated water	Ingestion
Parenteral oxygen/Ozone gas mixture	Intramuscular
	Subcutaneous
	Intratonsilar
	Intrathecal
	Intraperitoneal
	Periganglionic
	Intraforaminal
	Paravertebral
	Intra-articular
	Intradiscal
	Oral submucous
	Supralaminar
	Epidural
	Penile
	Intravenous
Parenteral ozonated water	Intra-tumoral
Routes systemic	Minor Autohemotherapy (MiAH)
	Extracorporeal blood oxygenation and ozonation (EBOO)
	Major Autohemotherapy (MAH)
	Intravenous
	Rectal insufflation (RI)
Topical application ozonated water	Wound wash
	Ozonized balneotherapy
	Mouthwash
	Nasal irrigation
	Sinus irrigation
	Otological irrigation
	Vaginal irrigation
	Intrauterine irrigation
	Intestinal irrigation
	Intravesical irrigation
	Ozone sauna therapy
Topical oxygen/Ozone gas mixture	Otological insufflation
	Intrafistula insufflation
	Hyperbaric bagging
	Intrauterine insufflation
	Intravesical insufflation
Topical ozonated oil	Embrocation
	Inhalation
Topical ozonated saline solution	Wound wash
	Mouthwash
	Sinus irrigation
	Otological irrigation
	Vaginal irrigation
	Intrauterine irrigation
	Intravesical irrigation

We developed a characterization matrix to synthesize the findings. This matrix included: Full Text Citation; Interventions; Outcomes Group; Outcomes; Effect; Population; Database ID; Focus Country; Publication Country; Publication Year; Study Design. The search and analysis of documents was performed in April 2022.

## 3. Results

**Figure 1:**
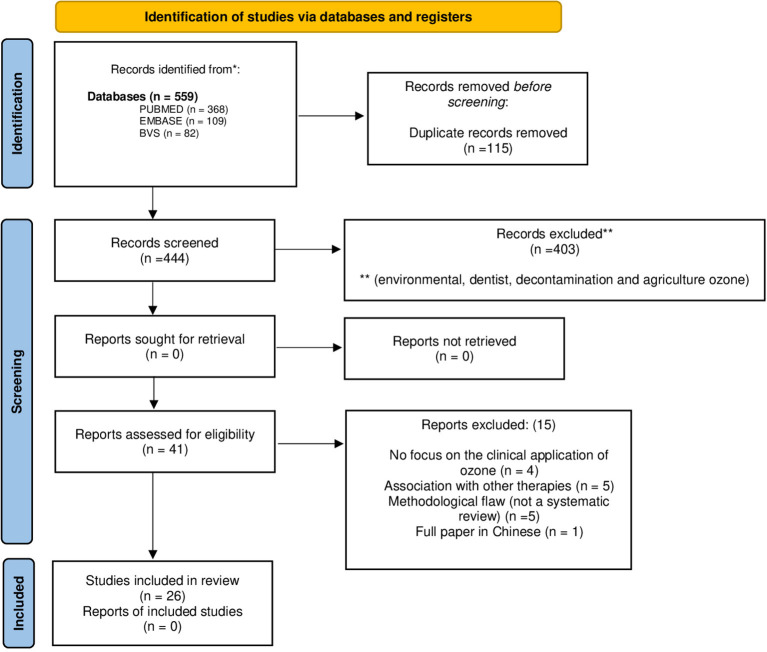
Ozone treatment literature flow according PRISMA flow diagram.

Until April 4th, 2022, the bibliographic search on treatment with ozone with application of the “SR” resulted in 559 records at databases (Pubmed, BVS and Embase). After excluding duplicates and analyzing the titles, abstracts and full texts, 26 studies (qualitative analysis) were included in the treatment with ozone EGMs (Figure 1).

### 3.1. Study design

The 26 SR included were: 18 SR, 1 SR with meta-analysis, 3 SR of randomized controlled trials, 3 meta-analyses, and 1 scoping review. Studies included were designed as with the majority being randomized clinical trials (16). Another 8 reviews are from observational clinical studies, and another 2 reviews are from non-randomized clinical trials.

The SR used in our work were performed Spain (2006 and 2019), USA (2009, 2019 and two in 2021), Brazil (2012, two in 2013, 2018 and 2019), China (2015), Iran (2018 and 2020), Australia (2018), Portugal (two in 2018), Mexico (2019), Switzerland (2021), England (2018, 2019 and 2021), The United Kingdom (2018, 2021 and 2022) and Italy (2021).

The primary studies included in SR are concentrated in European and Asian countries, other studies are from the United States, Brazil and Cuba. Five SR did not inform the countries of the primary studies.

### 3.2. AMSTAR evidence level

The AMSTAR tool showed that of the 26 SR analyzed, 9 high-level reviews, 3 moderate-level reviews, 11 low-level reviews and 3 low-critical reviews. The majority of the evidence proved the efficacy of treatment with ozone applied to reduce pain (42 associations), low back pain (18) and improvement in physical function (19) stand out (Table 2).

**Table 2:** List of 26 systematic reviews related to ozone therapy, considering country of origin, level of confidence according to AMSTAR and database where it was published.

**References**	**Title**	**Study Topic**	**Country**	**AMSTAR confidence level**	**Database**
Cochrane (18)	Ozonioterapia no tratamento da úlcera crônica de membros inferiores: revisão sistemática de literatura	Wounds	Brazil	High	LILACS
Liu et al. (20)	Ozone therapy for treating foot ulcers in people with diabetes	Wounds	China	High	MEDLINE
Fitzpatrick et al. (21)	Ozone therapy for the treatment of chronic wounds: A systematic review	Wounds	Australia	Moderate	MEDLINE
Wen et al. (19)	A systematic review of ozone therapy for treating chronically refractory wounds and ulcers	Wounds	UK	High	MEDLINE
Leon et al. (22)	Risks of ozonated oil and ozonated water on human skin: A systematic review	Skin	UK	Low	MEDLINE
Costa et al. (23)	Ozonoterapia na Osteoartrose do Joelho: Revisão Sistemática / Ozone Therapy in Knee Osteoarthritis: A Systematic Review	Knee	Portugal	High	MEDLINE
Raeissadat et al. (24)	An investigation into the efficacy of intra-articular ozone (O2–O3) injection in patients with knee osteoarthritis: a systematic review and meta-analysis	Knee	Iran	High	MEDLINE
Arias-Vázquez et al. (25)	Short term therapeutic effects of ozone in the management of pain in knee osteoarthritis: A Meta-analysis	Knee	Mexico	High	MEDLINE
Noori-Zadeh et al. (26)	Intra-articular ozone therapy efficiently attenuates pain in knee osteoarthritic subjects: A systematic review and meta-analysis	Knee	UK	High	MEDLINE
Sconza et al. (27)	Oxygen-Ozone Therapy for the Treatment of Knee Osteoarthritis: A Systematic Review of Randomized Controlled Trials	Knee	USA	Low	MEDLINE
Arias-Vázquez et al. (28)	Efficacy of Ozone Infiltrations in the Treatment of Knee Osteoarthritis vs. Other Interventional Treatments: A Systematic Review of Clinical Trials	Knee	Spain	Low	MEDLINE
Oliviero et al. (29)	The temporal effect of intra-articular ozone injections on pain in knee osteoarthritis	Knee	England	Low	MEDLINE
Hedayatabad et al. (30)	The Effect of Ozone (O3) vs. Hyaluronic Acid on Pain and Function in Patients with Knee Osteoarthritis: A Systematic Review and Meta-Analysis	Knee	Iran	Low	MEDLINE
Li et al. (31)	Intra-articular oxygen-ozone vs. hyaluronic acid in knee osteoarthritis: A meta-analysis of randomized controlled trials	Knee	England	High	MEDLINE
Carmona et al. (32)	Revisión sistemática: Ozonoterapia en enfermedades reumáticas/Ozone therapy in rheumatic diseases: A systematic review	Herniated Disc/Low Back Pain	Spain	Low	MEDLINE
Steppan et al. (12)	A metaanalysis of the effectiveness and safety of ozone treatments for herniated lumbar discs	Herniated Disc/Low Back Pain	USA	Moderate	MEDLINE
De Oliveira Magalhaes et al. (33)	Ozone Therapy as a Treatment for Low Back Pain Secondary to Herniated Disc: A Systematic Review and Meta-analysis of Randomized Controlled Trials	Herniated Disc/Low Back Pain	Brazil	Low	MEDLINE
Cochrane (18)	Ozonioterapia no Tratamento da dor lombar: Revisão sistemática da literatura	Herniated Disc/Low Back Pain	Brazil	High	LILACS
Costa et al. (34)	Ozone therapy for low back pain. A systematic review	Herniated Disc/Low Back Pain	Portugal	Moderate	MEDLINE
Sampaio et al. (35)	A utilizacao da Ozonioterapia no tratamento da lombalgia associada a hernia de disco lombar	Herniated Disc/Low Back Pain	Brazil	Critically Low	LILACS
de Andrade et al. (36)	Efetividade da ozonioterapia comparada a outras terapias para dor lombar: revisão sistemática com matanálise de ensaios clínicos randomizados	Herniated Disc/Low Back Pain	Brazil	High	MEDLINE
Rimeika et al. (37)	Metanalysis on the effectiveness of low back pain treatment with oxygen-ozone mixture: Comparison between image-guided and non-image-guided injection techniques	Herniated Disc/Low Back Pain	England	Low	MEDLINE
Sconza et al. (38)	Oxygen-ozone therapy for the treatment of low back pain: A systematic review of randomized controlled trials	Herniated Disc/Low Back Pain	Italy	Low	MEDLINE
Najadir Cristina De Faria et al. (39)	Efficacy of Ozone Therapy in the Treatment of Tinnitus: A Systematic Review	Tinnitus	USA	Low	MEDLINE
Radvar et al. (40)	Using Ozone Therapy as an Option for Treatment of COVID-19 Patients: A Scoping Review	COVID-19	USA	Critically Low	MEDLINE
Baeza-Noci and Pinto-Bonilla (41)	Systemic Review: Ozone: A Potential New Chemotherapy	Cancer	Switzerland	Critically Low	MEDLINE

### 3.3. Outcomes and effects

Reviews evaluated the effect of some Ozone interventions: parenteral oxygen/ozone gas mixture; parenteral ozonated water; systemic routes; topical application ozonated water; topical oxygen/ozone gas mixture; and topical ozonated oil. We found a higher number of evidences in the parenteral application of the oxygen-ozone gas mixture, followed by local and topical application.

The interventions were associated with 55 health outcomes divided into 7 groups: cancer, infection, inflammation, pain, quality of life, wound healing and adverse effects.

In total, there were 334 associations between intervention and outcome, with emphasis on the interventions of the parenteral mixture oxygen/ozone gas (192 associations, 57%).

A positive [154] or potentially positive (23) effects were reported in the interventions/outcomes. Inconclusive effect was reported for 132 associations and no effect was reported for 8 associations. The effects of treatment with ozone were: 14 positive, 5 potential positive, 5 inconclusive and 2 no effect.

**Figure 2:**
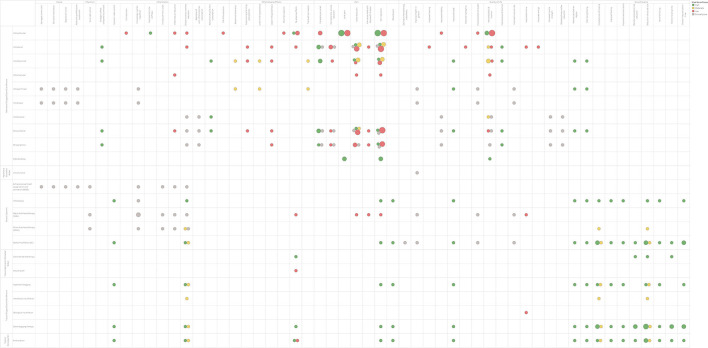
Ozone treatment Evidence and Gaps Map.

The Figure 2 provides a broad visual view of the evidence base of treatment with ozone. The bubble chart describes the estimated volume of research based on the number of treatment with ozone SR included in the largest review, summarizing the routes of administration and the outcomes related to inflammation, pain, wound healing, quality of life and adverse events. The Table 3 show the other information related to SR as the total number of patients, the population studied, the methodology and the results of treatment with ozone in evidence-based medicine.

**Table 3:** Compilation of information related to the final 26 SR include reference, country, sample number, population studied, methodology and results/conclusions.

**References**	**Country**	**Patients number**	**Population studied**	**Methodology**	**Results and conclusions**
Cochrane (18)	Brazil	190	>18 years old with chronic lower-limbs ulcer	SR according to the Cochrane methodology. Randomized clinical trials that tested OT or associated with placebo or other active treatment option were included.	Three studies were included, one of OT in ischemic ulcer and two for diabetic ulcers. The studies were heterogeneous, making it impossible to perform meta-analyses. There is poor methodological evidence that OT may be effective and safe in the treatment of chronic ulcers related to DM and peripheral arterial insufficiency. No evidence was found on the effectiveness of OT in the treatment of venous ulcers.
Liu et al. (20)	China	212	Any age with DM and foot ulcers	Search databases: COCHRANE WOUNDS GROUP SPECIALIZED REGISTRY, COCHRANE CENTRAL REGISTRY OF CONTROLLED TRIALS (CENTRAL), OVID MEDLINE (IN PROCESS AND OTHER UNINDEXED CITATIONS), OVID EMBASE, EBSCO CINAHL, SCIENCE WOUNDS CITATION INDEX, CHINESE BIOMEDICAL LITERATURE DATABASE and THE CHINESE CLINICAL REGISTRY. Without restrictions on the language, date, or configuration of the study. RCT comparing OT with placebo or any other interventions for foot ulcers in people with DM. The methodological quality of the included studies and the level of evidence of the results were evaluated using cochrane's bias risk tool and the GRADE (Evaluation of classification, development and evaluation) approach, respectively.	Three studies were included in the review. The overall risk of polarization was high for two trials. No side effects were observed. OT was associated with a greater reduction in ulcer area from baseline to the end of the study than treatment with antibiotics (DM −20.54 cm^2^, 95% CI −20.61–−20.47), and a shorter duration of hospitalization (MD −8.00 days, 95% CI −14.17–−1.83), but did not appear to affect the number of ulcers healed over 20 days (RR 1.10, 95% CI 0.87–1.40). The other two trials (*n* = 111) comparing OT plus usual care with usual care for foot ulcers in people with DM. The meta-analysis showed no evidence of a difference between groups for the outcomes of reduction of ulcer area (DM −2.11 cm^2^, 95% CI −5.29–1.07), the number of ulcers healed (RR 1.69, 95% CI 0.90–3.17), adverse events (RR 2.27, 95% CI 0.48–10.79), or amputation rate (RR 2.73, 95% CI 0.12, 64.42). The available evidence was three small RCT with unclear methodology, so we cannot draw reasoned conclusions about the efficacy of OT for foot ulcers in people with DM.
Fitzpatrick et al. (21)	Australia	453	Any age with chronic wounds	Search databases: GOOGLE SCHOLAR, PUBMED, COCHRANE LIBRARY and reference lists. English language studies, RCT and trials that reported the use of OT in the topical treatment of chronic wounds were included. The level of bias and quality of the studies were evaluated.	Nine studies were selected and submitted to meta-analysis. There was a significant improvement in the wound with OT, especially in the treatment of chronic wounds. Compared to standard treatment, OT can improve the proportion of chronic wounds healed in a shorter period of time, and further scientific research is needed.
Wen et al. (19)	UK	1.055	Wound of second- or third-degree actinic ulcers following a radiotherapy cycle, chronic venous leg ulcers, digital ulcers in systemic sclerosis, critical limb ischemia and diabetic foot ulcers	Search databases: COCHRANE LIBRARY, PUBMED, OVID EMBASE, WEB OF SCIENCE and CHINESE BIOMEDICAL LITERATURE. RCTs about participants with chronic wounds were included. Risk of bias assessment was performed by the Cochrane risk-of-bias tool. A randomized-effects model was applied to pool results according to the types of wounds or ulcers.	Twelve included studies, ozone was implemented by topical application (ozone gas bath, ozonated oil, ozone water flushing) and systematic applications including autologous blood immunomodulation and rectal insufflation. Compared with standard control therapy for diabetic foot ulcers, OT regardless of monotherapy or combined control treatment markedly accelerated the improvement of the wound area SMD = 66.54%, 95% CI = [46.18, 86.90], *P* < 0.00001 and reduced the amputation rate RR = 0.36, 95% CI = [0.24, 0.54], *P* < 0.00001. But there is no improvement in the proportion of participants with completely healed wounds and length of hospital stay. No adverse events associated with OT have been reported. The efficacy of OT for other wound types is still uncertain because of no sufficient studies. More high-quality randomized controlled trials are needed to confirm the efficacy and safety of OT for chronic wounds or ulcers.
Leon et al. (22)	UK	2.628	Ozonated oil and water on skin	Search databases: WEB OF SCIENCE CORE COLLECTION, EMBASE, COCHRANE LIBRARY, OVID MEDLINE(R) < 1946 to September 2020>, and GOOGLE SCHOLAR up to September 2020. Studies included about human cells, tissues, or patients who had ozonated water or oil applied topically. Required outcomes included any evaluation of risk of damage to skin tissue. The authors extracted information and results from the studies. Additionally, the authors evaluated the risk of bias relative to our desired outcomes using the Cochrane collaboration's risk of bias tool.	Nine studies were included in this review. Two studies evaluated the cytotoxicity of ozonated liquids on human cells, five studies evaluated ozonated liquids in RCTs, one was a post-market surveillance study, and one was a crossover study in humans. None of the included studies found any significant human dermatologic risks associated with ozonated water or liquid. Because of the small sample size, however, additional short- and long-term RCTs specifically designed to evaluate the dermatological risks of ozonated liquids are recommended.
Costa et al. (23)	Portugal	493	Elderly patients with mild to moderate knee OA	Search databases: PUBMED, EMBASE, COCHRANE LIBRARY, SCOPUS and WEB OF SCIENCE. The English descriptors ‘ozone therapy' and ‘knee OA'. were used.	Six randomized and controlled studies were included. Few studies reported mild adverse effects associated with the procedure. OT was efficient in the short term, in relation to placebo and when combined with HA. It is important that new RCT assess the benefits/risks of OT both in the short and medium/long term.
Raeissadat et al. (24)	Iran	428	Age of 64 years with knee OA	A SR of three large databases (PUBMED, COCHRANE CENTRAL REGISTER OF CONTROLLED TRIALS and GOOGLE SCHOLAR) was conducted to identify all RCT that evaluated the efficacy of intra-articular ozone injection vs. a control injection.	Five randomized clinical trials were included. The efficacy of intra-articular ozone injection was significantly higher than placebo and slightly lower than other control injections with no significant difference. Ozone may be recommended as an efficient, durable non-surgical treatment for at least 3–6 months in the mild or moderate treatment of knee OA.
Arias-Vázquez et al. (28)	Mexico	718	>18 years with clinical and radiographic diagnosis of OA	Only randomized clinical trials evaluating the efficacy of intra-articular or periarticular ozone infiltrations in the treatment of knee OA in humans were included. The meta-analysis was performed according to the declaration of preferred report.	Eight studies were selected. OT had a therapeutic effect when compared to placebo or other noninvasive treatments. No significant effects were found in favor of OT when compared to the use of HA or PRP. However, the use of ozone had a significant short-term benefit, reducing knee pain. The benefits of pain relief lasted between 3 and 6 months. Intra-articular ozone infiltrations can be used as an optional effective treatment for the treatment of knee OA related pain. There are short-term effect benefits that peak in about 1 month of treatment, with a gradual decline in efficacy after 3 to 6 months of treatment. Further studies are needed to improve our understanding about the efficacy of this treatment.
Noori-Zadeh et al. (26)	UK	419	Aged between 30 and 85 years with OA	Search databases: PUBMED, MEDLINE, GOOGLE SCHOLAR, SCOPUS and EMBASE without restriction of start date until July 2018.	10 studies were included for meta-analysis. Analysis of Q and I^2^% indices showed a high heterogeneity in the selected studies (2600.330 and 99.654, respectively). The primary analysis for the main hypothesis found that the weighted pooled effect size for the impact of intra-articular OT for pain reduction was as follows: SMD = −28.551 (95% confidence interval, −32.553–−24.549). The *P*-value for the significance of the combined SMD examined by the z-test was 0.000 considered statistically significant. The meta-analysis evidenced that intra-articular OT is an effective way for chronic pain management in OA.
Sconza et al. (27)	USA	858	Patients performed a radiographic assessment before treatment and were classified according to Kellgren-Lawrence (K-L) grades. Five papers included patients with radiographic K-L grades II-III. 4 papers included patients with radiographic K-L grades I-II. One paper included grades I-III.	A SR was performed on PUBMED, COCHRANE, EMBASE, RESEARCHGATE and PEDRO, with the inclusion criteria: (1) RCT, (2) written in English language, (3) published on indexed journals in the last 20 years (1998-2018), (4) dealing with the use of ozone intra-articular injection for the treatment of OA. The risk of bias was assessed by the Cochrane Risk of Bias tool for RCT.	Eleven studies were included. Patients in the control groups received different treatments: placebo in 1 trial; HA in 2 studies; HA and PRP in 1 trial; corticosteroids in 4; and hypertonic dextrose, radiofrequency, or celecoxib + glucosamine in the remaining 3 trials. None of the studies included reached “good quality” standard, 2 studies were ranked as “fair,” and the other were considered “poor.” No major complications or serious adverse events were reported following intra-articular OT, providing pain relief at short term. On the basis of the available data, no clear indication emerged from the comparison of OT with other established treatments for OA. The analysis of the available RCT on OT for OA revealed poor methodologic quality, with most studies flawed by relevant bias, thus severely limiting the possibility of drawing conclusions on the efficacy of OT compared with other treatments. However, on the basis of the data, OT proven to be a safe approach with effects in pain control and functional recovery in the short-middle term.
Arias-Vázquez et al. (28)	Mexico	781	Patients with clinical and radiographic diagnosis of OA, age older than 18, and comparison of pain score and/or self-evaluated function	Search databases: PUBMED DIALNET, SCIELO databases and other electronic sources such as Google Scholar, using a search period from January 2000 to May 2018. Only RCTs that used a therapeutic intervention with Hypertonic Dextrose Prolotherapy vs other substances infiltration or some other performance interventional procedure for treating patients with knee OA.	Ten studies were included. In terms of pain reduction and function improvement, prolotherapy with hypertonic dextrose was more effective than infiltrations with local anesthetics, as effective as infiltrations with hyaluronic acid, ozone or radiofrequency and less effective than PRP and erythropoietin, with beneficial effect in the short, medium and long term. In addition, no side effects or serious adverse reactions were reported in patients treated with hypertonic dextrose. Although HDP seems to be a promising interventional treatment for knee OA, more studies with better methodological quality and low risk of bias are needed to confirm the efficacy and safety of this intervention.
Oliviero et al. (29)	England	353	> 18 years or older with symptomatic knee OA and a minimum 4-week follow-up	Search databases: PUBMED, EMBASE, GOOGLE SCHOLAR and SCOPUS (to January 2019) was performed to define the effect on the knee after injections of ozone, on pain and physical function. In patients with OA. For data synthesis across studies, the primary outcome was evaluated by comparing the results of the VAS and the arthritis index of the WOMAC.	Six RCT were included. The data analyzed shows that the most promising protocol in reducing pain is an intra- articular injection of 20 μg/mL × 20 mL of O_3_, once a week for four consecutive weeks. However, appropriately powered studies with sufficiently long follow-up are necessary to establish which adequate dose(s) and frequency of administration. Data synthesis showed that intra-articular ozone injections reduce knee pain and total WOMAC scores. OT produces an improvement of the symptoms at 1, 3, and 6 months after treatment, although such improvement is not always statistically significant. At 12 months, however, the evaluation scales have returned to the initial scores. No severe adverse events were recorded.
Hedayatabad et al. (30)	Iran	467	Patients with knee OA	Search databases: PUBMED/MEDLINE, ISI WEB OF KNOWLEDGE, COCHRANE LIBRARY and SCOPUS. All RCT that compared the effects of intra-articular injection of ozone vs. HA on pain in adult patients with arthritis were used.	Of 6 studies, 4 were in English, 1 was in Persian, and 1 was in German language. The overall SMD for VAS pain did not show a significant difference between the groups although it favored HA injection (1.27 [95%CI: (−0.12)−2.66]). Total WOMAC score showed a significant difference over the time favoring HA injection [4.5 [95%CI: 1.1–8]]. However, no single time point showed any significant difference between groups. This meta-analysis showed no significant difference between HA and ozone in reducing pain and improving function in patients with knee OA, although the overall results favored HA over ozone. Since previous studies have shown comparable results between HA and placebo, ozone seems to fall in the same category with more placebo effect rather than a real disease-modifier.
Li et al. (31)	England	289	>18 years diagnosed with end-staged knee OA	Search databases: PUBMED, EMBASE, WEB OF SCIENCE and THE COCHRANE LIBRARY. High quality RCTs comparing HA with oxygen-ozone in the treatment of knee OA were selected. Statistical heterogeneity for each RCT were assessed using Chi2 test and the I2 statistic. Quality assessment was performed by using Cochrane Collaboration's tool.	The meta-analysis indicated that there was a significant difference between groups regarding stiffness and function in the VAS and WOMAC. The improvements in WOMAC pain were similar. No significant difference in adverse events occurrence was observed. Intra-articular injection of HA was associated with a significantly reduction in VAS score at 1st month compared to oxygen-ozone. And there were significant differences in WOMAC stiffness, and function at 6-month follow-up between groups.
Carmona (32)	Spain	3006	Patients with musculoskeletal diseases and/or Raynaud syndrome. Not described age.	Search databases: PUBMED, EMBASE and COCHRANE LIBRARY. All studies that demonstrated the efficacy or effectiveness of OT in any musculoskeletal disease were selected.	Six relevant studies were included, 5 in lumbar disc herniation and 1 in Raynaud's syndrome. There was great variability in the injected ozone dose, as well as in the controls used for comparison. All results were subjective and there was no blind evaluation of the results. The study of Raynaud's syndrome included only 4 patients. Adverse effects were not evaluated in detail. Therefore, the use of OT in musculoskeletal diseases is based on poor quality studies. Currently, data not support an adequate risk/benefit for OT in rheumatic diseases.
Steppan et al. (12)	USA	7902	Age between 13 and 94 years old with herniated disc	The meta-analysis method of random effects was used to estimate the results of treatment with OT in herniated discs. Search databases: PUBMED and THE INTERNATIONAL JOURNAL OF OZONE THERAPY WEBSITE. Separate meta-analyses were performed for VAS, ODI and modified MacNab results scale, as well as for complication rate.	Twenty studies were included in the meta-analyses. Meta-analyses were performed on the results of OT treatment in 8,000 patients from multiple centers. Treatment with OT in herniated discs is an effective and extremely safe procedure. The estimated improvement in pain and function is impressive, given the broad inclusion criteria. Pain and function results are similar to the results of lumbar discs treated with surgical discectomy, but the complication rate is much lower (< 0.1%) recovery time is significantly shorter.
De Oliveira Magalhaes et al. (33)	Brazil	Randomized studies: 876 Observational Studies: 6699	> 18 years, having low back pain due to lumbar disc herniation or degenerative disc disease	Search databases were consulted from 1966 to September 2011. The quality of articles was based on cochrane review criteria modified for randomized studies and criteria of the Health Research and Quality Agency.	Eight observational studies were included, and four trials randomized in the meta-analysis. The level of evidence for a indicated for long-term pain relief was II-3 for OT applied intradiscally and II-1 for paravertebrally. The classification of the recommendation was 1C for intradiscal OT and 1B for paravertebral OT. OT seems to produce positive results and low morbidity rates when applied percutaneously in the treatment of chronic low back pain.
Centro Cochrane do Brasil (42)	Brazil	1263	> 18 years diagnosed with nonspecific low back pain or lumbosciatalgia	A SR was carried out according to the Cochrane Collaboration methodology. Including RCT that tested OT alone or associated with placebo or other treatment option.	Eight randomized clinical trials were included. There is a great heterogeneity between the studies, which made it difficult to perform meta-analysis. There is evidence of long-term superiority of OT compared to steroid injection, radiofrequency, and open surgery. Further studies with appropriate methodology and comparison of OT to placebo procedures are needed, as well as studies comparing the various doses and means of ozone application.
Costa et al. (34)	Portugal	2422	Patients with lumbar disc herniation between 18 and 65 years	Search databases: PUBMED and SCOPUS, followed by a three-step selection process. The data were processed by two independent reviewers and the information was collected based on predefined variables. Articles in English; in ozone treatment were included.	Seven studies were included in the review. Ozone vs. placebo, ozone and global postural reeducation vs. global postural reeducation alone, ozone and steroid vs. steroid, ozone vs. steroid and, ozone vs. microdiscectomy were compared. All studies showed similar or better results in the experimental group, except in the study comparing ozone with micro-discectomy. Side effects have been reported in three studies (insomnia, itching, papules around the point of infiltration, gastritis, dizziness, tachycardia and hot flushes). The included studies reported an improvement in pain and functional scores.
Sampaio et al. (35)	Brazil	428	Patients with low back pain caused by herniated disc. Not described age.	PRISMA and PICOS were used to analyze the design of the studies. Search databases: PUBMED, CAPES and SCIELO.	Four clinical trials were selected. The efficiency of OT was confirmed as a therapeutic method in the reversal of pain symptomatology in patients with lumbar disc herniation. Therefore, OT associated with physical therapy treatment may contribute to the relief of pain associated with back pain.
Andrade et al. (36)	Brazil	958	> 18 years diagnosed with low back pain	RCT have been used to compare the efficacy of ozone and other therapies for the relief of low back pain. Search databases: MEDLINE, SCOPUS, LILACS AND EMBASE, from the beginning of the platform until 2018.	Six clinical trials were selected for SR and three for meta-analysis. OT is more effective in relieving low back pain; however, the studies showed a high or uncertain risk of bias. Meta-analysis on the efficacy of pain relief showed no significant difference between groups over the three-month period and showed greater efficacy of OT at 6 months compared to other therapies (steroid and placebo). Therefore, OT used for 6 months for relief of low back pain is more effective than other therapies; however, this result is not definitive, as data from studies with risk moderate to high risk of bias have been used.
Rimeika et al. (37)	England	not described	Patients with discogenic disease	Search databases: NATIONAL LIBRARY OF MEDICINE MEDLINE. Metanalysis was based on retrospective bibliometric search of papers on percutaneous image-guided and non-image-guided oxygen-ozone injections in the treatment of LBP and sciatica published between January 1980 and December 2020. Case reports, congress posters/abstracts, animal model studies, reviews/meta-analyses, as well as methodological or *ex-vivo* researches were not included.	In the 45 articles included in the metanalysis, ozone was administered as a gas mixture of O_2_-O_3_ with a concentration ranging between 10 and 40 μg/mL; OT injection was coupled to steroids in 28.3% cases, anti-inflammatory drugs in 2.2% cases and anesthetics in 13% cases, whereas in 6.5% cases was coupled to other techniques including radiofrequency thermocoagulation, collagenase injection, bioresonance magnetotherapy and/or electrostimulation. In conclusion, percutaneous oxygen-ozone injection is a minimally invasive, cost-effective, repeatable and highly available procedure for the treatment of lumbar disc herniation-related low back pain when poorly responsive to conservative treatments. Concerning the heterogeneity in analyzed results, the data argue in favor of the need for a higher methodological rigor in patients' selection and stratification when oxygen-ozone therapy for LBP treatment is envisaged. Further studies are still required to assess the superiority of this method compared to conventional surgery and different mini-invasive techniques both in terms of efficacy and results' stability over time.
Sconza et al. (38)	Italy	2597	Patients with LBP and herniated disc	Search databases: PUBMED and SCOPUS with the following inclusion criteria: (1) RCT, (2) published in the last 20 years, (3) dealing with OT in patients with LBP and herniated disc, (4) comparing the results of OT with those of other treatments. The risk of bias was assessed by the Cochrane Risk of Bias tool.	Patients in the control groups received different treatments, from oral drugs to other injections, instrumental therapy and even surgery: corticosteroids were used in 5 studies, analgesic therapy in 2 studies; placebo, microdiscectomy, laser-therapy, TENS and postural rehabilitation, percutaneous radiofrequency intradiscal thermocoagulation and compartmental block were tested in the other trials. None of the studies included reached “good quality” standard, 3 were ranked as “fair” and the other were considered “poor.” Comparison of OOT results with other approaches showed that, in the majority of studies, OOT was superior to the control treatment, and also when compared to microdiscectomy, ozone showed non inferiority in terms of clinical outcomes. No major complications or serious adverse events were reported. The injected O3 volume and concentration were widely different, ranging from 3 to 20 mL and from 10 to 60 μg/mL of concentration. OOT is a promising approach for LBP, with a good safety profile and therapeutic potential, and it could be included among the armamentarium of the conservative management of this common condition. Based upon the evidences, OOT provides better outcomes compared to local administration of CS and systemic drugs. Less clear is its efficacy against surgical procedures such as microdiscectomy.
De Faria et al. (39)	USA	113	Patients with tinnitus	Search databases: MEDLINE, WEB OF SCIENCE, COCHRANE LIBRARY, SCIENCE DIRECT, SCOPUS, GOOGLE SCHOLAR, EMBASE AND LILACS. Data was processed by two independent reviewers. Only SR, RCT, Observational Studies and Case Series published in English or Spanish with no date limit that evaluated the use of OT for the treatment of tinnitus were included. From 264 references retrieved after duplicates removal, 260 articles were excluded according to the pre-established selection criteria. The assessed articles were then subjected to risk of bias analysis, and one of them was excluded.	The concentration of ozone in the ozone and oxygen mixture was 8 mg/L; the flow rate was 60 mL/min. Inhalation was performed for 10 min per day once a day for 10 days. Their patients obtained improvements in vertigo, hearing loss, tinnitus and nystagmus of 90%, 80%, 65% and 100%, respectively. Therefore, the authors concluded that OT is effective for the treatment of peripheral vestibulocochlear syndrome. No side effects were observed. OT may be a potential treatment for tinnitus. High-level evidence, such as well-conducted RCT, are still needed to confirm the efficacy and safety of this therapy.
Radvar et al. (40)	USA	85	Patients with COVID-19	Search databases: MEDLINE (PUBMED), EMBASE, COCHRANE LIBRARY (CENTRAL), and TRIP were searched for evidence-based articles published until 6 April 2020. All studies and primary reports, which assessed the effects of OT on COVID-19 were included in this review regardless of study designs. Because of the novelty of the project and the small amount of available data, no exclusion criteria were applied to avoid losing data and maximize comprehensiveness. The search process was done independently by two reviewers. All 234 data from the initial search were screened by title and abstract. 72 studies were selected for further evaluation. Also, relative studies were included considering the concept of review and settled scopes. Nine studies for COVID-19 and 5 hypothetical reviews with expert opinions indicating potential effects of OT for the novel coronavirus were obtained.	According to the evidence it is postulated the OT is potentially effective in the treatment of the novel coronavirus due to the mechanism of actiion and the pathogenesis of the COVID-19. This SR considered data from the OT in COVID-19, however, lack of sufficient evidence and theoretical contents of studies may be limiting factors, therefore, it is suggested to perform more clinical trials.
Baeza-Noci and Pinto-Bonilla (41)	Switzerland	50	Patients with cancer	Search databases: PUBMED A SR was carried out following the PRISMA guidelines, using the terms 'ozone AND cancer' in the ‘title' field, finding 50 references. After a critical reading of the title and abstract, only 6 references fulfilled the scope (5 *in vitro* studies, 1 *in vivo* study). To expand the literature review, the authors performed a detailed reading of the references in the articles found in the first search. From the scan of this reference in these articles and also in the two review articles mentioned above, they found 17 more articles (6 *in vitro* studies, 8 *in vivo* studies, 3 clinical studies). As very different types of articles were found (pre-clinical controlled, pre-clinical uncontrolled, case series), a PRISMA checklist could not be fully performed, so it is not considered a strict systematic review, but rather an evidence review.	The only paper showed increased survival (30.5 months compared with the standard value of 11.9 months) in a series of four patients with recurrent glioblastoma that were treated with re-resection of the tumor (after relapsing) and intratumoral ozone administered monthly through a catheter (5 mL of oxygen–ozone gas at a concentration of 40 μg of ozone per mL of oxygen—a total dose of 200 μg each time), together with the standard protocol of chemotherapy. The patients received a median of 27 (range: 3–44) oxygen–ozone applications. Another patient was treated with ozone just after the first surgery (together, the RT and CT protocols used in all the patients were included in this case series); he is still alive and without recurrence after 53 months. Two side effects were reported; one catheter was removed temporarily because of an infection and another one, in a different patient, was removed because of a hemorrhage. Other clinical papers, mainly case reports or short case-control series, have suggested a collaborative effect of ozone with RT or CT. The oxygenating effect of ozone (10) can increase the radiosensitivity of some tumors, improving the survival rate.
					The modulation on the immune system induced by ozone (10) may play a role in enhancing the anti-cancer effect of other drugs. RCT should be the third step, after deeper preclinical studies have been done. To develop clinical studies, different interventional approaches can be used to dispense ozone into the tumor: specific arterial embolization, intratumoral injection, and catheterization. As a single application of ozone is not likely to affect all tumor cells, several applications of this gas would be needed to progressively affect all the tumor. Further studies testing the tumor cell reaction to ozone *in vivo* must be done to use ozone as a CT agent in a systemic way.

DM, Diabetes Mellitus; CI, Confidence Intervals; OT, Ozone Therapy; SMD, Standardized Mean Difference; RR, Risk Ration; HA, Hyaluronic Acid; PRP, Platelet-Rich Plasma; VAS, Visual Analog Scale; WOMAC, Western Ontario And Mcmaster Universities Arthritis Index; ODI, Oswestry Disability Index; LBP, Low Back Pain; CS, Corticosteroids; SR, Systemic Review; RT, Radiotherapy; CT, Chemotherapy; GRADE, Grading Of Recommendations Assessment, Development And Evaluation; RCT, Randomized Controlled Trials; PRISMA, Preferred Reporting Items For Systematic Re- Views And Meta-Analyses; OA, Osteoarthritis.

### 3.4. Cancer

For cancer a single review was included (41). Intratumoral ozone was administered monthly through a catheter (5 mL of oxygen-ozone gas at a concentration of 40 μg ozone per mL of oxygen - a total dose of 200 μg each time), along with the standard chemotherapy protocol, with a median of 27 oxygen-ozone applications. Two side effects were reported; one catheter was temporarily removed because of an infection and in another patient the catheter was removed because of hemorrhage.

The observed outcomes were: regression of the tumor; decrease of the metastases and of the proliferation of cancer cells; and damage to cancer cells.

Its effect was inconclusive, and the AMSTAR level was critically low, so further studies are needed to analyze this outcome.

### 3.5. Wound healing

This category included results from four reviews (18–21) that used the route of administration: rectal insufflation, bag, ozonized oil, Minor Autohemotherapy, topical ozonized blood and washing with ozonized water. This group was the second most evident, 61.18%, with the healing outcome being the most cited. The reviews analyzed, 2 had a positive effect (19, 21) and 2 inconclusive (18, 20). For the AMSTAR level of the reviews evaluated, three were rated high (18–20) and one moderate (21).

The observed outcomes were: complete wound healing; improved vascularization; improved of ischemia and reperfusion; relief of itching; cutaneous/subcutaneous infection reduction; reduction of scar adhesions; shorter healing time; shorter hospitalization time; bleeding reduction; erythema reduction; reduction in the number of hospitalization; member amputation risk reduction; reduction of wound size; shorter healing time; shorter hospitalization time; improved feeling.

The treatment with ozone improves wound healing and treatment time but does not seem to be superior to conventional treatments, but in chronic treatments, it can lead to healing in a shorter time, however additional studies are needed. The main evidences observed were: decrease in the size of injuries and pain; complete wound closure; improved capillary blood glucose; shorter hospital stay and reduction of oxidative stress markers. No adverse events associated with treatment the ozone were reported.

### 3.6. Infection

A single review was included (40). The systemic route was used to analyze the effect of treatment with ozone in patients with COVID-19. Its effect was inconclusive and the AMSTAR level was critically low, so further studies are needed to analyze this outcome.

The observed outcomes were: viral load control; sepse prevention; bacterial colony reduction; hepatic enzyme reduction; antibiotics usage time reduction.

Five studies were included in the review (40) and it was concluded that the potential role of systemic treatment with ozone is effective in controlling COVID-19 because of its antiviral, oxygenating, anti-inflammatory, oxidation-balancing, and immunomodulatory effects. However, there is not sufficient evidence to endorse the efficacy of treatment with ozone on novel coronavirus disease.

### 3.7. Inflammation

This category included results from eleven reviews (18, 21, 28, 31, 35–37, 39–42). For the outcome inflammation four routes of administration were used: topical oxygen-ozone gaseous mixture, topical ozonated oil, parenteral oxygen-ozone gaseous mixture and systemic. The reviews analyzed, 5 had a positive effect (21, 35–37, 42), 2 potentially positive (28, 39), 3 inconclusive (18, 40, 41) and 1 no effect (31), concluding therefore that treatment with ozone can be effective for the reduction of inflammation. For the AMSTAR level of the eleven reviews evaluated, four were rated high (18, 31, 36, 42) and three critically low (35, 40, 41).

The observed outcomes were: increase of nitric oxide; glycemic index control; Nrf2 induction; improved articular stiffness; inflammation reduction; reduction of painkillers consumption; reduction of anti-inflammatories consumption; edema reduction; fever reduction; IL-6 reduction; oxidative stress markers reduction; NFKb reduction; TNF-Alfa reduction; immunologic system regulation; analgesic effect compared to corticoid.

The pathologies that most benefited from the reduction of inflammation with treatment the ozone were OA (28, 31, 36), wounds (18, 21) and herniated disc/low back pain (35, 37, 42). The decrease in inflammation also occurred in the reviews of tinnitus (39), COVID-19 (40) and cancer (41), but with less effectiveness.

### 3.8. Pain

This category included results from nineteen reviews (12, 18, 23–38, 42). Most of the available evidence focused on the use of ozone for pain reduction, with 103.31% being evidenced and the highlighted outcomes were: pain reduction (42 associations), low back pain (18) and improvement in physical function (19).

The observed outcomes were: complete pain relief; headache; joint pain; wounds pain; lower back pain; muscle pain; neuropathic pain; pain reduction; herniated disc volume reduction; pain associated with raynaud's disease.

The reviews showed pain reduction for herniated disc/low back pain (7 reviews) and OA (9 reviews). Of the reviews analyzed, 11 had a positive effect (12, 23, 24, 26, 27, 34–38, 42), 4 potentially positive (25, 28, 29, 36), 2 inconclusive (18, 32) and 2 no effect (30, 31), concluding therefore that treatment with ozone can be effective for the reduction of rem disc hernia/low back pain and also OA. Of the nineteen reviews evaluated, seven SR were classified with a high (18, 23, 25, 26, 31, 36, 42) AMSTAR level and only one critically low (35) certifying this outcome.

In the most recent review (2021) (37) it was observed that percutaneous oxygen-ozone injection is a minimally invasive, cost-effective, repeatable and highly available procedure for the treatment of lumbar disc herniation-related low back pain when poorly responsive to conservative treatments. Furthermore, imaging-guided procedures showed a better therapeutic performance with higher impact on pain reduction and lower age-related variability.

### 3.9. Quality of life

This category included results from sixteen reviews (12, 18, 24, 27–31, 34–36, 38, 39, 41, 42). The second highest available evidence focused on the use of treatment with ozone for improve the quality of life, with 78.23% evidenced. Improved quality of life was observed in reviews of OA, herniated discs/low back pain, wounds, tinnitus and cancer. The most frequent administration routes were parenteral and systemic oxygen-ozone gaseous mixture.

The observed outcomes were: increased muscle strength; improved physical function; improved social function; improved mobility; improved health in general; improved vitality; improved physical aspect; improved mood; fatigue reduction; osteotendinous reflex reduction; wellbeing feeling; patient satisfaction degree; improved vertigo; improved tinnitus; improved nystagmus; improved hearing; decrease chemotherapy side effects; improved survival rate.

Of the analyzed reviews, nine had a positive effect (12, 24, 27, 34–36, 38, 42), three potentially positive (28, 29, 39), two inconclusive (18, 41) and two no effect (30, 31), concluding that treatment with ozone can be improve quality of life. In the AMSTAR level, it was seen that of the sixteen reviews evaluated, five were classified as high (18, 31, 34, 36, 42) AMSTAR, two moderate (12, 34), seven low (24, 27–30, 38, 39) and two critically low (35, 41). We believe that this result is due to the fact that this outcome is very subjective. Further studies that assess the quality of life of patients using validated scales should be performed.

### 3.10. Adverse effects

This category included results from ten reviews (12, 19, 22, 27–29, 31, 33, 38, 39) that only observed mild adverse effects in four studies (12, 29, 33, 38), such as: abdominal distension, lower limb hypoesthesia, lower limb paresthesia, transient worsening of pain, mild exacerbation reaction and fainting sensation. In the other 6 review no adverse effects were observed.

The reviews analyzed, 5 had a positive effect (12, 19, 22, 27, 38), 4 potentially positive (28, 29, 33, 39) and 1 no effect (31), concluding that treatment with ozone can be safe. However, in the AMSTAR level, it was seen that of the ten reviews evaluated, two were classified as high (19, 31) AMSTAR, one moderate (12) and seven low (22, 27–29, 33, 38, 39). More studies should be carried out, as well as the creation of protocols for the correct execution of the technique.

The only route of administration that demonstrated these adverse effects was the parenteral oxygen-ozone gaseous mixture. The works reported adverse events of transient paresthesia and secondary infection, probably due to an inadequate asepsis procedure. Treatment with ozone can be considered an option to treat low back pain related to herniated lumbar disc that did not respond to conservative treatment, representing an alternative to surgery. However, future studies are needed to demonstrate whether the effects of treatment with ozone persist over time.

The review (22) included nine studies. Two studies specifically evaluated the cytotoxicity of ozonated liquids on human cells, five studies evaluated ozonated liquids in randomized controlled trials, one was a post-market surveillance study, and one was a crossover study in humans. None of the included studies found significant human dermatological risks associated with ozonated liquid.

## 4. Discussion

This treatment with ozone EGMs is based on 26 studies and provides a broad overview of available evidence of complementary therapies in reduction of pain, lower back pain and improvement of physical function, healing, reduction of inflammation and infection. It shows the volume of available research and highlights areas where the interventions showed potential positive and positive effects.

In Brazil, the Federal Council of Dentistry (CFO) recognized treatment with ozone as a dental procedure in all areas of modern dentistry through CFO Resolution no. 166/2015. On October 18, 2017, the Federal Senate unanimously approved Bill No. 227, which authorizes the prescription of treatment with ozone throughout the national territory. This federal bill is currently being processed in the Chamber of Deputies under no. 9001/2017. During the 1st International Congress of Integrative Practices and Public Health (INTERCONGREPICS), the Ministry of Health of Brazil recognized and included treatment with ozone as an integrative and complementary practice of the SUS through the MS Ordinance No. March 2018. On December 9, 2021, the Constitution and Justice Committee (CCJ) of the Chamber of Deputies unanimously approved the opinion on Bill No. 9,001/17, which authorizes the use of treatment with ozone in health, in a complementary way, throughout the national territory.

The experience is considered a safe practice from the practical guidelines and the most developed practices from experiences and treatment protocols. The toxic effects by inhalation in high doses of ozone gas, for humans and animals, for the bronchi and lungs are well known. A review of the literature on ozone genotoxicity and cytogenetic studies administered intramuscularly, intraperitoneally, intratesticularly and rectally performed *in vivo* in laboratory animals has shown mainly good results. The absence of genotoxic effects in rats has also been documented in the bone marrow and sperm of animals treated with ozone at medicinal doses. The cases on safety issues of treatment with ozone discussed in international journals allow us to state that most safety problems are secondary to infections or traumatic infections due to malpractice. Commonly, the ozone molecule itself is not responsible for severe reactions (12, 43–48).

Treatment with ozone promotes stimulation of oxygen metabolism (49), modulation of the immune system (50), antimicrobial action (51), tissue repair (52) and hemorrheological improvement (52, 53).

In injured tissues, treatment with ozone favors cell recovery, contributing to the reduction of edema, inflammation, oxygenation of ischemic areas (54) and restoration of blood flow in collateral vessels (53). Also promoting the increase in the epithelialization process, increasing matrix deposition and cell proliferation. Lesions treated with the oxygen-ozone mixture demonstrate the formation of granulation tissue with fewer inflammatory cells, a greater number of myofibroblasts, greater collagen deposition and an increase in the number of blood vessels. Myofibroblasts and macrophages from ozone-treated skin lesions showed increased expression of Fibroblast Growth Factor (FGF). This increased expression of FGF results in differentiation, activation and proliferation of fibroblasts and myofibroblasts associated with angiogenesis. These findings corroborate the perception that treatment with ozone favors collagen production, neovascularization and tissue remodeling (52, 55).

It is also known that treatment with ozone, through reactive oxygen species, interacts with exudates from lesions, causing ozone degradation into peroxides, stimulating tissue repair and improving oxygenation in the affected area. It favors platelet aggregation in injured tissues, increasing the endogenous production and release of these growth factors (56, 57).

The antimicrobial properties of ozone have a broad spectrum, against viruses, bacteria, fungi, yeasts and protozoa, especially in aqueous media. The mechanism of antibacterial action of ozone is through the oxidation of unsaturated lipids constituting the cytoplasmic membrane. After exposure to ozone, the olefinic bonds are attacked to form an ozonide. This action initiates the destruction of the cell's functional capacity and may even be sufficient to cause the death of weaker cells (44). This ozonide has a high oxidation potential, is unstable and exerts its own disinfecting action by attacking enzymes, sulfhydryl groups, or aldehydes, releasing peroxyl compounds, which are also disinfectants. Finally, the cell is lysed and the cytoplasm is dispersed. Thus, the action of ozone is characterized by the increase of many other oxidizing substances that can compete or complement the action of ozone to destroy critical sites within the cell or generally to oxidize protoplasm. This ripple effect is unique to ozone and its decomposition products. Virus envelopes provide complex cell attachment, penetration, and egress strategies. Peplomers, fine-tuned to adjust to changing receptors on a variety of host cells, constantly assemble a new configuration of glycoproteins under the direction of parts of the viral genome, adapting to the host cell's defenses. Envelopes are fragile and can be broken by medical ozone and its derivatives (peroxides, aldehydes, hydroxyperoxides). Viruses involved with lipids in aqueous media are readily inactivated by ozone through the oxidation of their envelope lipoproteins and glycoproteins (51).

There are also several studies on the effectiveness of ozone in general (58) and specific viral infections: herpes (59), hepatitis B and C (60), HIV (50), poliovirus (61), hepatitis A and norovirus (62), rotavirus (63). Ozone can inactivate viruses by direct oxidation of its components *in vitro*. However, virucidal activity *in vivo* becomes uncertain when viruses are in biological fluids or when they are intracellular (pneumocytes, hepatocytes, epithelia, CD4+ lymphocytes, monocytes, glial and neuronal cells), because the powerful antioxidant system protects viral integrity (64).

Medical ozone modulates the Nrf2 system (65, 66) producing three effects. First, it normalizes the redox balance through the increase of antioxidants in the cytoplasm, mitochondria and finally in the plasma, mainly glutathione peroxidase, but also glutathione reductase, NADPH and Superoxide Dismutase (SOD). Second, it induces the production of HemeOxygenase-1 (HO-1), a protective enzyme, together with heat shock proteins such as HSP-60, HSP-70 and HSP-90 (66). Third, it activates the NF-κB system, which modulates the production of pro-inflammatory interleukins in injured tissues (67). These effects contribute to restoring the normal functioning of inflamed tissues and decreasing the amount of plasma interleukins.

The treatment with ozone is also capable of inducing an adaptation to oxidative stress or promoting oxidative preconditioning by increasing and preserving antioxidant systems (68–70). The adaptation is developed after multiple exposures to treatment with ozone (71). Prolonged exposure to treatment with ozone in elderly individuals causes an increase in both ATP and 2,3-DPG in red blood cells (72). The oxygen-ozone mixture has the function of restoring and improving the metabolism of oxygen, sugars and fats to produce energy, through the normal metabolic pathways of controlled combustion: improvement of glycolysis, the respiratory chain, the fatty acid cycle, glucose 6-phosphate dehydrogenase, pyruvic acid decarboxylation (49) and increased intraerythrocytic glutathione levels (73, 74).

EGMs is created and published to include graphically presenting the best evidence found, analyzed and classified, and linking it to bibliography and full research text to make the information more accessible (14). In wounds the treatment with ozone improves wound healing and reduces treatment time but does not seem to be superior to conventional treatments. In chronic treatments, it can lead to healing in a shorter time, but additional studies are needed. In knee joints, ozone is effective in reducing pain up to 3 months with effects reducing after 3 months. Adverse effects are rarely reported. However, further studies are needed regarding the standardization of protocols for clinical practice. In herniated discs, ozone is effective and present low risk mainly *via* paravertebral. The intradiscal route has also been shown to be effective whether administered with or without steroids. There is evidence of long-term superiority of treatment with ozone compared to steroid injection, radiofrequency and open surgery.

Among the strengths of this study, we highlight that it was the first evidence map for the use of treatment with ozone in general therapeutic areas of our knowledge, including a comprehensive search of eligible SR, with explicit inclusion and exclusion criteria. The study provides readers with an overview, as well as detailed information, of evidence and gaps in medical ozone treatment, as well as access to primary studies.

As limitations we mention that we do not calculate the effect sizes as in a meta-analysis, nor do we provide risk assessments of bias, but we overcome these limitations by relying on the author's skills in conducting and evaluating the quality of the studies, choosing the results, analyzing the results effects and susceptibility to publication and results report bias. We suggest that in future studies, health and economic impact assessments for health services should be adopted, as well as robust methodologies for evaluating clinical trials.

## 5. Conclusions

This treatment with ozone EGMs proved that ozone can be effective in controlling pain, infections, inflammation and wound healing, as well as increasing the quality of life with high evidence level. No serious adverse effects were observed. The parenteral application of the oxygen-ozone gas mixture was the administration route more effective, followed by local and topical routes. Therefore, treatment with ozone can be offered as another option in the treatment of several clinical conditions, as an integrative treatment, considering its low cost, efficiency and safety. The EGMs brought confidence and credibility to the prescription and use of treatment with ozone.

## Data availability statement

The datasets presented in this study can be found in online repositories. The names of the repository/repositories and accession number(s) can be found in the article/Supplementary material.

## Author contributions

MS, ML, and AA: conceptualization. CM: methodology, software, and formal analysis. MS and AA: validation. MS, JB-N, CB, and AA: investigation and writing–review and editing. MS, CB, and AA: resources and writing–original draft. MS, CM, CB, and AA: data curation. MS, JB-N, ML, CM, CB, and AA: visualization. CB and AA: supervision. MS: project administration and funding acquisition. All authors contributed to the article and approved the submitted version.
