# Supplementary material for: The role of ozone treatment as integrative medicine. An evidence and gap map
Source: Front Public Health. 2023 Jan 16;10:1112296. doi: 10.3389/fpubh.2022.1112296 (PMC9885089; doi:10.3389/fpubh.2022.1112296)
Supplement: Supplementary file 1 [file Data_Sheet_1.DOCX]

**Supplementary files**

**Appendix S1 – List of studies included in the Evidence Map**

Andrade RR, Oliveira-Neto OB2, Barbosa LT3, Santos IO4, Sousa-Rodrigues CF5, Barbosa FT6. Rev Bras Anestesiol. 2019 Sep 11. pii: S0034-7094(18)30585-3. doi: 10.1016/j.bjan.2019.06.007. [Epub ahead of print] <http://rba.elsevier.es/pt-efetividade-da-ozonioterapia-comparada-outras-articulo-S0034709418305853>

Arias-Vázquez PI, Tovilla-Zárate CA, Bermudez-Ocaña DY, Legorreta-Ramírez BG, López-Narváez ML. Eficacia de las infiltraciones con ozono en el tratamiento de la osteoartritis de rodilla vs. otros tratamientos intervencionistas: revisión sistemática de ensayos clínicos [Efficacy of Ozone Infiltrations in the Treatment of Knee Osteoarthritis Vs Other Interventional Treatments: A Systematic Review of Clinical Trials]. Rehabilitacion (Madr). 2019 Jan-Mar;53(1):43-55. Spanish. doi: 10.1016/j.rh.2018.11.001.

Arias-Vázquez PI, Tovilla-Zárate CA, Hernández-Díaz Y, et al. Short term therapeutic effects of ozone in the management of pain in knee osteoarthritis: A Meta-analysis. 2019. http://dx.doi.org/10.1002/pmrj.12088. <https://www.ncbi.nlm.nih.gov/pubmed/30689297>

Baeza-Noci J, Pinto-Bonilla R. Systemic Review: Ozone: A Potential New Chemotherapy. Int J Mol Sci. 2021 Oct 30;22(21):11796. doi: 10.3390/ijms222111796.

Carmona L. Revisión sistemática: ozonoterapia en enfermedades reumáticas TT - OT in rheumatic diseases: a systematic review. Reum clín. 2006;2(3):119-123. <https://www.sciencedirect.com/science/article/pii/S1699258X06730327>

Centro Cochrane do Brasil. Ozonioterapia no Tratamento da dor lombar: revisão sistemática da literatura. 2013. [unpublished] <https://cajaclin.com.br/wp-content/uploads/2019/05/21-ozonioterapia-no-tratamento-da-dor-lombar.pdf>

Centro Cochrane do Brasil. Ozonioterapia no tratamento da úlcera crônica de membros inferiores: revisão sistemática de literatura. 2013. [unpublished] <https://cajaclin.com.br/wp-content/uploads/2019/09/COCHRANE-Rev.-Sistem%C3%A1tica-Oz%C3%B4nio-e-%C3%9Alceras-2013.pdf>

Costa T, Linhares D, Ribeiro da Silva M, Neves N. OT for low back pain. A systematic review. TT - OT for low back pain. A systematic review. Acta Reum Port. 2018;43(3):172-181. <http://actareumatologica.pt/article_download.php?id=1331>

Costa T, Rodrigues-Manica S, Lopes C, et al. Ozonoterapia na Osteoartrose do Joelho: Revisão Sistemática. TT - [OT in Knee Osteoarthritis: A Systematic Review]. Acta Med Port. 2018;31(10):576-580. <http://dx.doi.org/10.20344/amp.10330>. <http://actareumatologica.pt/article_download.php?id=1331>

Fitzpatrick E, Holland OJ, Vanderlelie JJ. OT for the treatment of chronic wounds: A systematic review. Int Wound J. 2018;15(4):633-644. doi:10.1111/iwj.12907 <https://doi.org/10.1111/iwj.12907>

Gc NCF, Vc VG, M SB, Jr FB. Efficacy of Ozone Therapy in the Treatment of Tinnitus: A Systematic Review. Int Tinnitus J. 2022 Mar 3;25(2):149-153. doi: 10.5935/0946-5448.20210027.

Javadi Hedayatabad, J., Kachooei, A. R., Taher Chaharjouy, N., Vaziri, N., Mehrad-Majd, H., Emadzadeh, M., Abolghasemian, M., & Ebrahimzadeh, M. H. (2020). The Effect of Ozone (O3) versus Hyaluronic Acid on Pain and Function in Patients with Knee Osteoarthritis: A Systematic Review and Meta-Analysis. The archives of bone and joint surgery, 8(3), 343–354. https://doi.org/10.22038/abjs.2020.46925.2292

Leon BR, Romary DJ, Landsberger SA, Bradner KN, Ramirez M, Lubitz RM. Risks of ozonated oil and ozonated water on human skin: A systematic review. Int Wound J. 2022 Jan 27. doi: 10.1111/iwj.13760.

Li Q, Qi X, Zhang Z. Intra-articular oxygen-ozone versus hyaluronic acid in knee osteoarthritis: A meta-analysis of randomized controlled trials. Int J Surg. 2018 Oct;58:3-10. doi: 10.1016/j.ijsu.2018.08.007.

Liu J, Zhang P, Tian J, et al. OT for treating foot ulceSR in people with diabetes. Cochrane database Syst Rev. 2015;(10):CD008474. doi:10.1002/14651858.CD008474.pub2. <https://doi.org/10.1002/14651858.CD008474.pub2>

Magalhaes FNDO, Dotta L, Sasse A, et al. OT as a treatment for low back pain secondary to herniated disc: a systematic review and meta-analysis of randomized controlled trials. Complement Ther Med. 2012;15(4):534-548. doi:10.1016/j.jvir.2009.12.393. <https://www.painphysicianjournal.com/current/pdf?article=MTU5Mg%3D%3D&journal=66>

Noori-Zadeh A, Bakhtiyari S, Khooz R, Haghani K, Darabi S. Intra-articular OT efficiently attenuates pain in knee osteoarthritic subjects: A systematic review and meta-analysis. Complement Ther Med. 2019;42:240-247. doi:10.1016/j.ctim.2018.11.023. https://www.sciencedirect.com/science/article/pii/S0965229918307337?via%3Dihub

Oliviero A, Giordano L, Maffulli N. The temporal effect of intra-articular ozone injections on pain in knee osteoarthritis. Br Med Bull. 2019 Dec 11;132(1):33-44. doi: 10.1093/bmb/ldz028. PMID: 31602459.

Radvar S, Karkon-Shayan S, Motamed-Sanaye A, Majidi M, Hajebrahimi S, Taleschian-Tabrizi N, Pashazadeh F, Sahebkar A. Using Ozone Therapy as an Option for Treatment of COVID-19 Patients: A Scoping Review. Adv Exp Med Biol. 2021;1327:151-160. doi: 10.1007/978-3-030-71697-4_12.

Raeissadat SA, Tabibian E, Rayegani SM, Rahimi-Dehgolan S, Babaei-Ghazani A. An investigation into the efficacy of intra-articular ozone (O2-O3) injection in patients with knee osteoarthritis: a systematic review and meta-analysis. J Pain Res. 2018;11:2537-2550. doi:https://dx.doi.org/10.2147/JPR.S175441. <https://www.ncbi.nlm.nih.gov/pubmed/30498370>

Rimeika G, Saba L, Arthimulam G, Della Gatta L, Davidovic K, Bonetti M, Franco D, Russo C, Muto M. Metanalysis on the effectiveness of low back pain treatment with oxygen-ozone mixture: Comparison between image-guided and non-image-guided injection techniques. Eur J Radiol Open. 2021 Dec 6;8:100389. doi: 10.1016/j.ejro.2021.100389.

Sampaio N da R, Cruz LRO, Medrado AP. A utilização da Ozonioterapia no tratamento da lombalgia associada à hérnia de disco lombar ¬ Uma Revisão Sistemática TT - Ozonetherapy in the treatment of Low Back Pain associated to Lumbar Disk Herniation ¬ A systematic review. Rev Pesqui Fisioter. 2018;8(4):579-587. <https://www5.bahiana.edu.br/index.php/fisioterapia/article/view/2107/2164>

Sconza C, Leonardi G, Kon E, Respizzi S, Massazza G, Marcacci M, Di Matteo B. Oxygen-ozone therapy for the treatment of low back pain: a systematic review of randomized controlled trials. Eur Rev Med Pharmacol Sci. 2021 Oct;25(19):6034-6046. doi: 10.26355/eurrev_202110_26881.

Sconza C, Respizzi S, Virelli L, Vandenbulcke F, Iacono F, Kon E, Di Matteo B. Oxygen-Ozone Therapy for the Treatment of Knee Osteoarthritis: A Systematic Review of Randomized Controlled Trials. Arthroscopy. 2020 Jan;36(1):277-286. doi: 10.1016/j.arthro.2019.05.043.

Steppan J, MeadeSR T, Muto M, Murphy KJ. A metaanalysis of the effectiveness and safety of ozone treatments for herniated lumbar discs. J Vasc Interv Radiol. 2010;21(4):534-548. doi:10.1016/j.jvir.2009.12.393. <https://www.jvir.org/article/S1051-0443(09)01668-6/fulltext>

Wen Q, Liu D, Wang X, Zhang Y, Fang S, Qiu X, Chen Q. A systematic review of ozone therapy for treating chronically refractory wounds and ulcers. Int Wound J. 2022 May;19(4):853-870. doi: 10.1111/iwj.13687.

**Appendix S2 – List of excluded studies**

*Reason for exclusion: Does not focus on clinical ozone therapy*

Al-Moraissi EA, Conti PCR, Alyahya A, Alkebsi K, Elsharkawy A, Christidis N. The hierarchy of different treatments for myogenous temporomandibular disorders: a systematic review and network meta-analysis of randomized clinical trials. Oral Maxillofac Surg. 2021 Oct 21. doi: 10.1007/s10006-021-01009-y.

Fliefel R, Tröltzsch M, Kühnisch J, Ehrenfeld M, Otto S. Treatment strategies and outcomes of bisphosphonate-related osteonecrosis of the jaw (BRONJ) with characterization of patients: a systematic review. Int J Oral Maxillofac Surg. 2015;44(5):568-585. <http://dx.doi.org/10.1016/j.ijom.2015.01.026>.

Shen L, Yuan T, Chen S, Xie X, Zhang C. The temporal effect of platelet-rich plasma on pain and physical function in the treatment of knee osteoarthritis: systematic review and meta-analysis of randomized controlled trials. J Orthop Surg Res. 2017;12(1):16. doi:10.1186/s13018-017-0521-3. <https://josr-online.biomedcentral.com/articles/10.1186/s13018-017-0521-3>

Sridharan K, Sivaramakrishnan G. Interventions for oral lichen planus: A systematic review and network meta-analysis of randomized clinical trials. Aust Dent J. 2021 Sep;66(3):295-303. doi: 10.1111/adj.12835.

*Reason for exclusion: Association with other therapies*

Anil U, Markus DH, Hurley ET, Manjunath AK, Alaia MJ, Campbell KA, Jazrawi LM, Strauss EJ. The efficacy of intra-articular injections in the treatment of knee osteoarthritis: A network meta-analysis of randomized controlled trials. Knee. 2021 Oct;32:173-182. doi: 10.1016/j.knee.2021.08.008.

Daste C, Laclau S, Boisson M, Segretin F, Feydy A, Lefèvre-Colau MM, Rannou F, Nguyen C. Intervertebral disc therapies for non-specific chronic low back pain: a systematic review and meta-analysis. Ther Adv Musculoskelet Dis. 2021 Jul 16;13:1759720X211028001. doi: 10.1177/1759720X211028001.

Naja M, Fernandez De Grado G, Favreau H, Scipioni D, Benkirane-Jessel N, Musset AM, Offner D. Comparative effectiveness of nonsurgical interventions in the treatment of patients with knee osteoarthritis: A PRISMA-compliant systematic review and network meta-analysis. Medicine (Baltimore). 2021 Dec 10;100(49):e28067. doi: 10.1097/MD.0000000000028067.

Nickles, M. A., Lio, P. A., & Mervak, J. E. (2022). Complementary and Alternative Therapies for Onychomycosis: A Systematic Review of the Clinical Evidence. Skin Appendage Disorders, 8(4), 269-279. <https://doi.org/10.1159/000521703>

Wen B, Wang Y, Zhang C, Xu W, Fu Z. Efficacy of different interventions for the treatment of postherpetic neuralgia: a Bayesian network meta-analysis. J Int Med Res. 2020 Dec;48(12):300060520977416. doi: 10.1177/0300060520977416.

*Reason for exclusion: Methodological flaw, it is not a systematic review*

Acosta JCR, Moreno MMAC, Hurtado JDC, Delgado MMM, Sierra JMS, Hernández DRG. Ozonetherapy In The Treatment Of Nocturnal Tinnitus. Int Tinnitus J. 2022 Mar 3;25(2):169-171. doi: 10.5935/0946-5448.20210030.

Al Bedah AMN, Khalil MKM, Elolemy AT, Alrasheid MHS, Al Mudaiheem A, Elolemy TMB. OT in postgraduate theses in Egypt: systematic review. J Egypt Public Health Assoc. 2013;88(2):57-66. doi:10.1097/01.EPX.0000431630.91853.ce.

Ameli J, Banki A, Khorvash F, Simonetti V, Jafari NJ, Izadi M. Mechanisms of pathophysiology of blood vessels in patients with multiple sclerosis treated with ozone therapy: a systematic review. Acta Biomed. 2019 Sep 6;90(3):213-217. doi: 10.23750/abm.v90i3.7265.

Anzolin AP, Bertol CD. Ozonioterapia como terapêutica integrativa no tratamento da osteoartrose: uma revisão sistemática. Br J Pain. 2018;1(2):171-175. <http://www.scielo.br/scielo.php?pid=S2595-31922018000200171&script=sci_arttext&tlng=pt>

Megele R, Riemenschneider MJ, Dodoo-Schittko F, Feyrer M, Kleindienst A. Intra-tumoral treatment with oxygen-ozone in glioblastoma: A systematic literature search and results of a case series. Oncol Lett. 2018 Nov;16(5):5813-5822. doi: 10.3892/ol.2018.9397.

*Reason for exclusion: Full text in Chinese*

Y.-G. D, W.-J. W. Radiofrequency ablation combined with ozone in the treatment of lumbar disc herniation: A meta-analysis. Chinese J Tissue Eng Res. 2014;18(13):2096-2103. doi:10.3969/j.issn.2095-4344.2014.13.022. <http://www.embase.com/search/results?subaction=viewrecord&from=export&id=L607994237>
